# Retrograde intraluminal balloon occlusion for redo descending or thoracoabdominal aortic repair via left thoracotomy

**DOI:** 10.1016/j.xjtc.2025.102187

**Published:** 2025-12-13

**Authors:** Kyokun Uehara, Mikage Inada, Masatomo Hayashi, Taku Shirakami, Makoto Takehara, Hiroyuki Hara, Mamoru Hamuro, Takashi Tsuji, Yoshio Arai

**Affiliations:** a Department of Cardiovascular Surgery, Tenri Hospital, Nara, Japan; b Department of Cardiovascular Surgery, Kumamoto Chuo Hospital, Kumamoto, Japan

**Figure:**
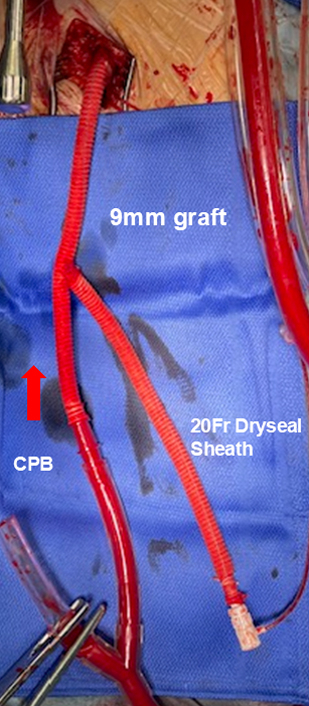
Y-graft enables CPB and retrograde balloon clamp in redo aortic surgery via thoracotomy.

**Central Message**

Retrograde intraluminal balloon occlusion avoids lung adhesiolysis and proximal descending aortic crossclamping, reducing operative complexity in redo descending or thoracoabdominal aortic surgery.

Redo descending or thoracoabdominal aortic repair via left thoracotomy after previous aortic surgery remains technically demanding. The major challenge is dense adhesion between the lung and aorta, particularly after open replacement, frozen elephant trunk (FET), or thoracic endovascular aortic repair (TEVAR, Figure 1, *A*).^1^ Adhesiolysis carries risks of pulmonary injury, bleeding, and respiratory failure.^2^ Proximal control is often difficult because of adhesions and perigraft thrombus, which preclude safe clamping (Figure 1, *B*). We describe a technique for safe proximal aortic control using a retrograde intraluminal occlusion balloon inserted through a Y-shaped composite graft during redo left thoracotomy. This study was approved by our institutional review board (no. 1372, May 23, 2023), and written informed consent for publication was obtained from all patients.

**Figure 1:**
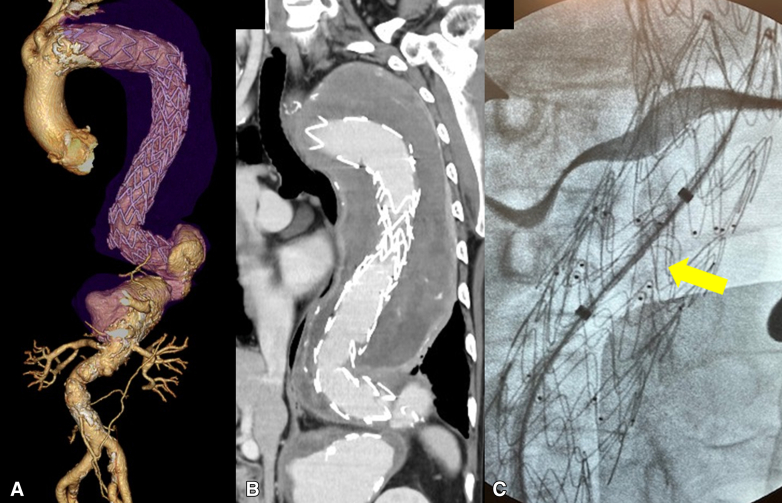
A, Thoracoabdominal aneurysm distal to stent graft. B, Proximal descending aorta with perigraft thrombus; no safe clamping site is available. C, Retrograde occlusion balloon positioned in stent graft (*arrow*).

## Surgical Technique

**Figure 2:**
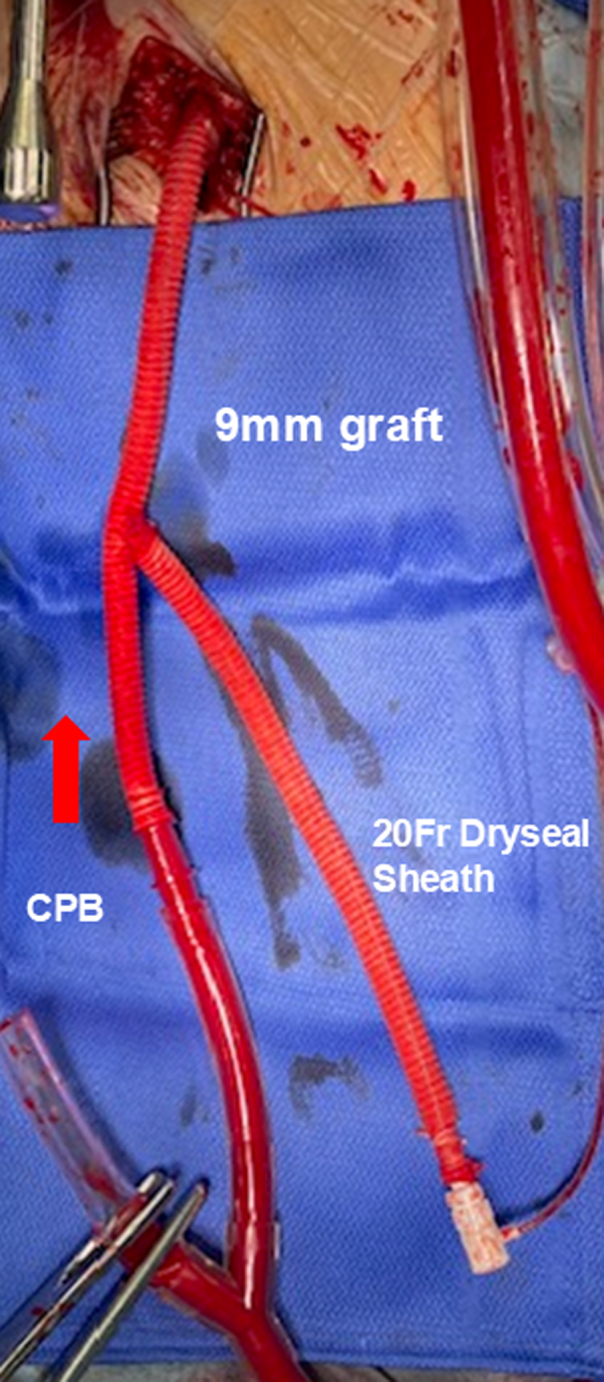
Y-shaped composite graft connected to cardiopulmonary bypass (*CPB*) and 20-Fr sheath for balloon insertion.

After careful left thoracotomy, the pleural cavity was opened under direct vision, avoiding injury to adherent lung tissue. Adhesions between the lung and aneurysmal aorta were left intact whenever possible. A 9-mm tube graft (J-graft; Japan Lifeline) was fashioned into a Y-shaped composite femoral graft. One limb was connected to the cardiopulmonary bypass (CPB) circuit, and the other limb served as the access route for balloon insertion. The composite graft was anastomosed to the femoral artery through an open groin incision after systemic heparinization. (Figure 2). A 20-Fr GORE DrySeal sheath (Gore Medical) was inserted into the second limb, and a 40-mL occlusion balloon (Tokai Medical Products) was advanced retrogradely under fluoroscopic and transesophageal echocardiography guidance into the proximal prosthetic graft, FET, or stent graft (Figure 1, *C*). Before initiating CPB, a brief test occlusion was performed by slowly inflating the balloon until the contralateral femoral arterial waveform disappeared. The balloon was then deflated during cooling. After the target temperature was reached, the balloon was reinflated to achieve proximal control. Transesophageal echocardiography was used to confirm the balloon's stability, and the catheter was firmly secured externally to prevent migration. After cooling, proximal occlusion was achieved via balloon inflation. Distal control was obtained by clamping the aorta together with the balloon shaft. The aneurysmal aorta was opened distal to the balloon, and proximal anastomosis was performed safely without dissecting adhesions. The balloon was withdrawn before completing the proximal suture line, followed by standard distal anastomosis (Video 1).

## Results

**Figure 3:**
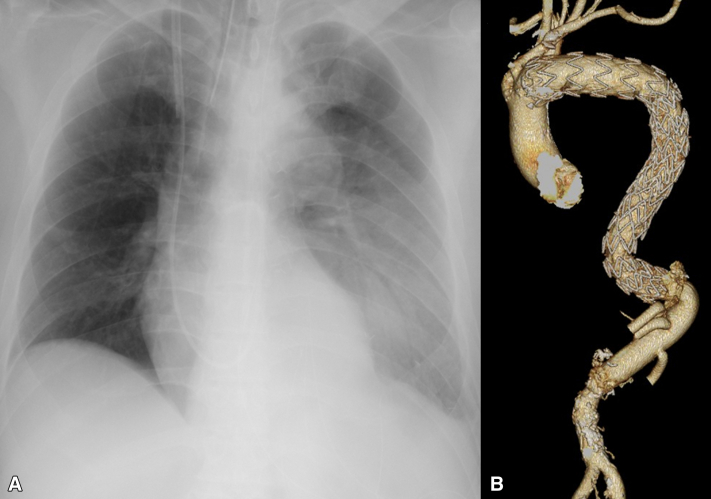
A, Immediate postoperative radiograph of the chest showing preserved lung expansion. B, Postoperative computed tomography scan confirming proximal anastomosis and graft continuity.

Four patients underwent redo descending or thoracoabdominal aortic replacement using intra-aortic balloon occlusion (Table 1). All had dissecting aneurysms after previous TEVAR, total arch replacement and FET, or descending aortic replacement. Proximal occlusion was achieved without technical complications or balloon dislodgment. No balloon ruptures occurred despite the presence of metallic struts in FET or TEVAR grafts. No major pulmonary injury or bleeding occurred (Figure 3, *A*). All patients were extubated within 1 to 3 days postoperatively and discharged uneventfully. Postoperative image confirmed satisfactory proximal anastomosis (Figure 3, *B*).

**Table 1:** Summary of 4 patients undergoing redo left thoracotomy with intra-aortic balloon occlusion

Patient	Original pathology	Age, y	Previous surgery	Operative procedure	Extubation time	Outcome
1	Chronic dissection (B)	71	TEVAR	TAAAR	Day 2	Discharged
2	Acute dissection (A)	74	TAR + FET, TEVAR	TAAAR	Day 1	Discharged
3	Chronic dissection (B)	52	DTAR	TAAAR	Day 3	Discharged
4	Acute dissection (A)	69	VSRR, TAR + FET	DTAR	Day 1	Discharged

*TEVAR*, Thoracic endovascular aortic repair; *TAAAR*, thoracoabdominal aortic aneurysm replacement; *TAR*, total arch replacement; *FET*, frozen elephant trunk; *DTAR*, descending thoracic aortic replacement; *VSRR*, valve-sparing aortic root replacement.

## Discussion

Redo descending or thoracoabdominal aortic repair via left thoracotomy is among the most complex cardiovascular procedures. Dense adhesions between lung and aortic grafts complicate exposure, increasing the risk of pulmonary injury and bleeding.^1^^,^^2^ Our retrograde intra-aortic balloon technique enables safe proximal control without adhesiolysis or crossclamping of the proximal descending aorta. Proximal control of the aneurysm was obtained by balloon occlusion, whereas distal control near the distal anastomotic site was achieved by applying an aortic clamp that included the balloon shaft. Compared with antegrade balloon insertion, the retrograde approach avoids arch manipulation and potential cerebral embolism. Left femoral anastomosis provides stable arterial inflow while permitting balloon access through the same graft. In our series, a 20-Fr sheath was selected because a smaller sheath (eg, 14 Fr) may allow leakage around the sheath in the 9-mm tube graft during surgery. A 40-cc Cook Coda balloon (Cook Medical) is also a feasible alternative. The Tokai balloon was chosen in this series because no balloon rupture was observed during our procedures. A critical component is preventing balloon migration; the catheter must be firmly secured at the DrySeal sheath exit site to ensure stability during CPB. This is essential to maintain reliable proximal control and prevent procedural complications. A previous report showed that intra-aortic balloon occlusion simplified thoracoabdominal repair after FET by avoiding deep hypothermic circulatory arrest.^3^ Our results suggest an additional advantage—avoidance of pulmonary injury during redo left thoracotomy. All patients achieved early extubation with preserved lung expansion, and no tracheostomy was required. This suggests that minimizing adhesiolysis contributes to smoother postoperative recovery. This technique is limited to cases in which the occlusion balloon can be placed within an existing graft or stent graft; use in native aorta is contraindicated because of rupture risk.

## Conclusions

Intra-aortic balloon occlusion through a Y-shaped composite graft provides safe and effective proximal control during redo descending or thoracoabdominal aortic repair via left thoracotomy. This technique avoids the need for adhesiolysis of the lung and surrounding tissues, as well as crossclamping of the proximal descending aorta, reduces operative complexity, and may facilitate early recovery in high-risk redo descending or thoracoabdominal aortic surgery.

## Conflict of Interest Statement

The authors reported no conflicts of interest.

The *Journal* policy requires editors and reviewers to disclose conflicts of interest and to decline handling or reviewing manuscripts for which they may have a conflict of interest. The editors and reviewers of this article have no conflicts of interest.

## Supplementary Data

Video 1 Retrograde intraluminal balloon clamping for proximal aortic control during redo aortic surgery via left thoracotomy. Video available at: https://www.jtcvs.org/article/S2666-2507(25)00568-1/fulltext.
