# Supplementary figures and images for: Retrograde intraluminal balloon occlusion for redo descending or thoracoabdominal aortic repair via left thoracotomy
Source: JTCVS Tech. 2025 Dec 13;35:102187. doi: 10.1016/j.xjtc.2025.102187 (PMC12881775; doi:10.1016/j.xjtc.2025.102187)

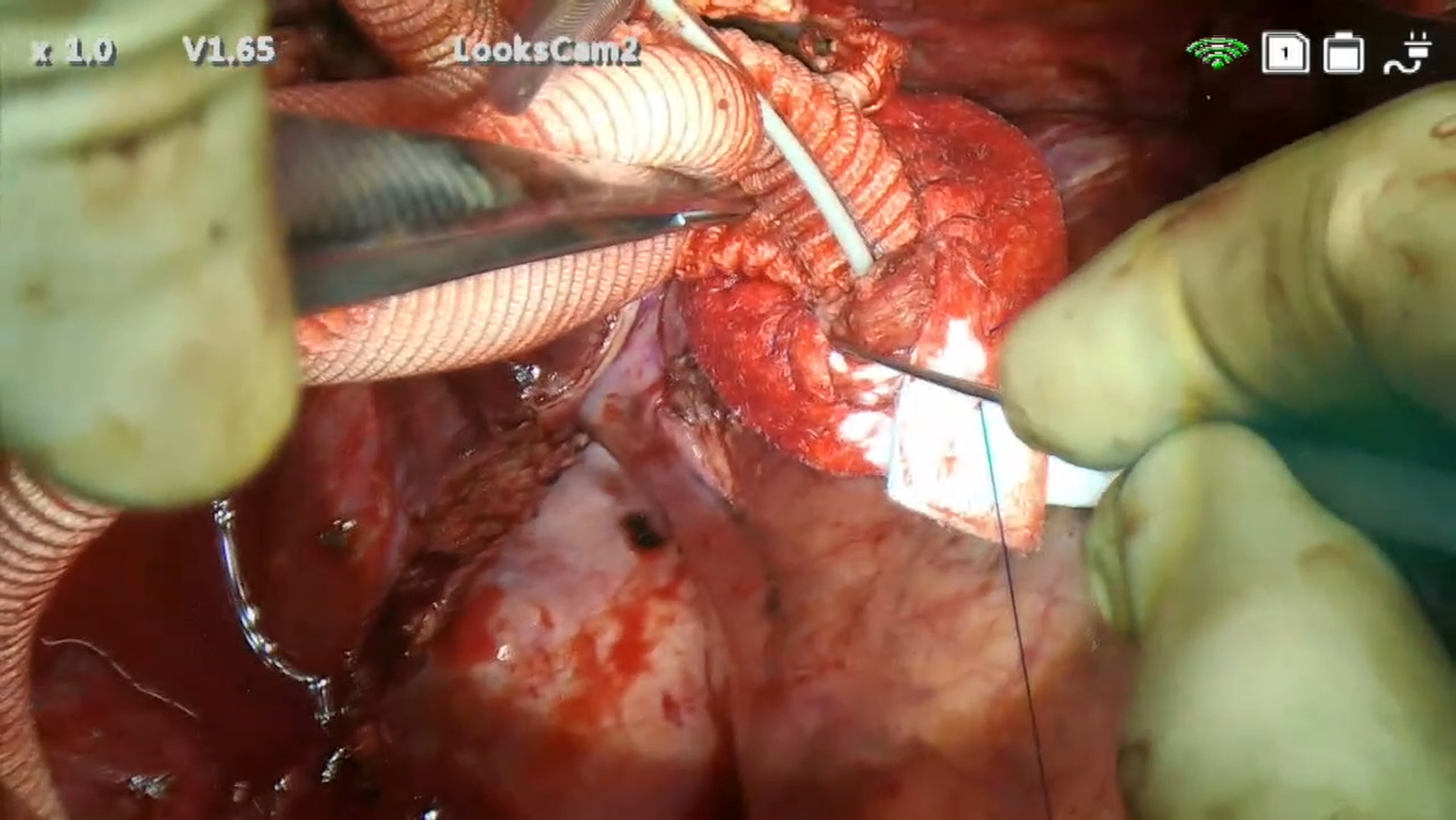

Supplement: Video 1 — Retrograde intraluminal balloon clamping for proximal aortic control during redo aortic surgery via left thoracotomy. Video available at: https://www.jtcvs.org/article/S2666-2507(25)00568-1/fulltext. [file fx2.jpg]
